# A study on plant disease and pest detection and counting based on multi-scale enhancement and cross-scale fusion

**DOI:** 10.3389/fpls.2026.1850591

**Published:** 2026-06-26

**Authors:** Junshu Wang, Li Liang, Yue Pan, Shuo Wang, Yueran Zhu, Wenlong Lyu, Shuai Zhao

**Affiliations:** 1 School of Artificial Intelligence, Guilin University of Electronic Technology, Guilin, China; 2 Guangxi Guangye Guitang Sugar Group Co., Ltd., Guigang, China; 3 Shenzhen International Graduate School, Tsinghua University, Shenzhen, China; 4 Guangxi Institute of Water Resources Research, Nanning, China; 5 Guangxi Key Laboratory of Water Engineering Materials and Structures, Nanning, China; 6 School of Business, Sun Yat-sen University, Guangzhou, China

**Keywords:** cross-scale attention fusion, multi-scale feature enhancement, object counting, plant disease and pest detection, precision agriculture

## Abstract

This paper targets the practical needs of plant disease and pest object detection and counting in complex field environments and proposes a lightweight improved framework based on YOLOv10. A MEMBA-F multi-scale feature enhancement module is introduced on the Neck to strengthen representations of small targets and weak-texture lesions, and a CSCAF cross-scale context-aware fusion module is designed to adaptively align high-level semantics with low-level details via cross-scale attention and gated selection, suppress background interference, and improve localization stability. The proposed method is systematically compared with two-stage detectors, YOLO-series models, and Transformer-based detectors on three public datasets, and is further investigated through ablation studies, confusion matrix analysis, and Grad-CAM interpretability analysis. In addition, a density-binned counting evaluation is conducted to validate robustness from sparse to dense scenarios. Experimental results demonstrate that the proposed method achieves superior performance in Precision, Recall, mAP@50, and mAP@50-95, and significantly reduces counting errors in dense scenes under deployable inference cost, providing reliable support for precision plant protection monitoring and decision making.

## 1 Introduction

Among these key factors, plant diseases and pests play an important role in affecting crop yield and quality. These diseases and pests have their own characteristics, including strong concealment, fast development, and irregular spatiotemporal distribution, which can even result in reduced crop yield or even complete crop failure. With the development trend of modern agriculture toward large-scale and precision-oriented agricultural production, it is clear that traditional monitoring methods based on field scouting have limitations in efficiency, objectivity, and consistency, which makes it unable to meet the requirements of large-scale, continuous, and fine management in agricultural fields Singh et al. (2020); Dong et al. (2025a). Computer vision-based automated detection and counting technologies can quickly locate, identify, and quantify disease lesions or pest targets in real-world fields, which can provide quantitative evidence for disease grading, pest density estimation, precision pesticide application, and other agronomic decision-making processes Zhang et al. (2022); Wen et al. (2022).

While deep learning-based object detection has made remarkable progress in various vision tasks, the object detection of plant disease and pest scenarios still has many challenges to overcome Tang et al. (2023); Bai et al. (2024). The challenges include the fact that the lesions or pests are small in scale, weak in texture, diverse in shape, and easy to be overwhelmed by the complex textures of the leaves. At the same time, there are many distracting factors in the scene background, such as the texture of the leaves, the shadow, the soil, and the weeds. These factors will lead to false detection Wang et al. (2025); Li et al. (2025). Moreover, the density of the lesions or pests is high, which will lead to the problem of insufficient fusion of multi-scale features. As a result, the model will have the problem of duplicate detection. At the same time, the model will have the problem of meeting the balance of the detection speed and the complexity of the model Dong et al. (2025b). Although these challenges have been discussed in previous studies, they remain difficult to solve in practical field monitoring because small-target detection, dense-instance counting, background interference suppression, and deployable inference efficiency need to be considered simultaneously. Therefore, this study does not simply restate these known difficulties, but focuses on a unified detection-and-counting setting in which reliable instance localization and quantitative counting are jointly required for plant disease and pest monitoring.

In order to deal with these problems, in this paper, the Backbone Neck Head architecture of YOLOv10 is extended and customized feature enhancement and cross-scale fusion are proposed according to the features of small targets, weak textures, high density, and complex background in agricultural scenarios. A MEMBA-F multi-scale feature enhancement module is proposed in the Neck part. By using lightweight sequence modeling and multi-scale information interaction, the fine textures and abnormal responses are enhanced, and the detectability of small lesions and insect targets is improved. Moreover, a CSCAF cross-scale context-aware fusion module is proposed. By using cross-scale attention and adaptive selection, the high semantic information is matched with low-level details, and background noise and irrelevant texture information are suppressed Wang et al. (2023b). At the same time, the number of detected targets is used as the counting result, and the counting error is also verified in density-binned settings.

The main contributions of this work are summarized as follows.

1. An improved YOLOv10 framework for plant disease and pest detection and counting is proposed. It enhances discriminative representations for weak textures and small targets while maintaining end-to-end real-time inference capability.
2. Two modules, namely MEMBA-F multi-scale feature enhancement and CSCAF cross-scale context-aware fusion, are designed. A collaborative mechanism of multi-scale enhancement and adaptive fusion improves localization and classification stability under complex backgrounds and dense occlusions, and systematic ablations validate the contribution of each component.
3. A comprehensive multi-dataset evaluation protocol is established. Beyond standard detection metrics, density-binned counting error evaluation and visualization analysis are further introduced, providing more interpretable and practically grounded experimental evidence for quantitative monitoring and precision plant protection decision making in agricultural scenarios.

## 2 Related work

### 2.1 Leaf disease detection and lesion localization in field conditions

For leaf disease detection and lesion localization in the real field environment, there are fundamental issues related to illumination and background changes, leaf occlusion and overlapping, large diversity in lesion scale, and domain shifts across varieties and growth stages, etc., which jointly influence the stability and generalizability of leaf disease detection. With respect to these issues, there is a gradual shift in leaf disease detection methods from image-level recognition to pixel-level or object-level fine-grained leaf lesion localization, as well as enhanced data augmentation and fine-grained supervision in training to enhance the separability of lesion boundaries and tiny spots in complex scenarios. Shoaib et al. proposed a method for leaf lesion segmentation and recognition, in which they emphasized the importance of exploiting the structural information in the diseased leaf regions for enhancing the representation capability Shoaib et al. (2022). Alqahtani et al. emphasized the importance of enhancing the reliability of leaf disease detection in an integrated localization-recognition framework Alqahtani et al. (2023). Moreover, Rodríguez-Lira et al. compared several variants of the YOLO algorithm on several natural environment datasets, in which they emphasized the importance of finding a balance between accuracy and speed in leaf disease detection in the real field environment while considering false positives and false negatives in complex background scenarios Rodríguez-Lira et al. (2024). However, from the perspective of precision agriculture, Li et al. demonstrated the applicability and engineering feasibility of the leaf disease detection framework in the real field environment for fruit tree leaf diseases Li et al. (2024).

From the methodological perspective, it is common for recent studies to make use of lightweight detectors and multi-scale feature modeling in addressing the demands of real-time performance and recognition efficiency for small lesion targets, as well as employing the outputs of the detectors in lesion counting and quantification for disease monitoring and management decision-making. Nwaneto et al. presented the implementation of early identification of foliar diseases by means of an object detection formulation. This demonstrates the applicability of the object detection paradigm in addressing disease early warning systems Nwaneto et al. (2025). Xu et al. presented the validation of the applicability of a lightweight leaf disease detector based on the YOLOv5 algorithm in addressing efficiency demands on the deployment side while ensuring competitive accuracy Xu et al. (2025). Apart from the above, it is also seen that in addressing the demands of disease severity grading, the work by Bi et al. presented the implementation of disease severity grading in the deep learning framework. This demonstrates the emerging trend in the extension of the object detection paradigm from addressing the question of whether the object is diseased or not toward addressing the question of the severity of the disease Bi et al. (2025). Kaur et al. improved the performance of object recognition and localization in addressing multi-disease scenarios by means of augmentation Kaur et al. (2025).

### 2.2 Object detection based lesion counting and severity quantification

Lesion counting and severity quantification based on object detection methods usually consider lesions as detectable targets and obtain interpretable statistical features based on the number, scale, and spatial distribution of detected boxes, as well as their relative relationships with the leaf region. Pan et al. proposed a method based on combining Faster R-CNN and few-shot learning for leaf scorch severity estimation. This work showed the possibility of using bounding box information in severity estimation Pan et al. (2022). Zhao et al. also proposed a method based on Faster R-CNN with multi-scale feature fusion for multi-disease detection in greenhouse environments. This work showed the possibility of using object detection methods in extracting lesion candidates in complex imaging environments Zhao et al. (2022). Meanwhile, Pal et al. proposed a method based on object detection for agricultural scenarios. This work showed the possibility of using object detection methods in recognizing disease severity Pal and Kumar (2023). In field deployment scenarios with strong real-time requirements, Lin et al. proposed a method based on improved YOLOX-Tiny for leaf disease detection. This work showed the possibility of using object detection methods in improving the efficiency of statistical features Lin et al. (2023).

Regarding severity quantification strategies, existing studies often combine detection outputs with grading or density modeling, mapping object-level information to disease-level indicators to reduce the cost of pixel-wise fine annotations and improve interpretability. Xiong et al. proposed a disease recognition and grading model targeting low-resolution imagery and complex backgrounds, showing that explicit modeling and grading mechanisms are still needed to stably output severity indicators under degraded imaging and noise interference Xiong et al. (2024); Abulizi et al. introduced structural improvements within the YOLO family for leaf disease detection, offering a pathway to enhance the reliability of counting and intensity estimation by improving recall and localization accuracy for small lesions Abulizi et al. (2025); Zhang et al. further integrated lightweight detection with fine-grained severity quantification to achieve real-time detection and accurate quantification under field conditions, reflecting a growing trend toward an integrated pipeline of detection–counting–grading Zhang et al. (2025); additionally, Rezaei et al. alleviated the annotation bottleneck for severity estimation from the perspective of automating pixel-level annotation, providing a useful complement for obtaining fine-grained supervision signals at lower cost and thereby improving consistency between detection and quantification Rezaei et al. (2026).

## 3 Methods

### 3.1 Problem definition

Given a leaf image dataset collected in natural field environments, $D={\left\{(I_{i},B_{i})\right\}}_{i=1}^{N}$, where $I_{i}\in {\mathbb{R}}^{H\times W\times 3}$ denotes the i-th RGB image and $B_{i}={\left\{b_{ij}\right\}}_{j=1}^{n_{i}}$ is the set of annotated lesion objects in this image. Each bounding box $b_{ij}=(x_{ij},y_{ij},w_{ij},h_{ij},c_{ij})$ is parameterized by the center coordinate $\left(x_{ij},y_{ij}\right)$, width and height $\left(w_{ij},h_{ij}\right)$, and a class label $c_{ij}\in \{1,\ldots ,C\}$, where C is the number of lesion categories and $n_{i}$ is the number of lesion instances in $I_{i}$. Our goal is to learn a joint detection-and-counting model f(·;Θ) that, given an input image I, outputs a set of predicted boxes $\hat{B}={\left\{({\hat{x}}_{k},{\hat{y}}_{k},{\hat{w}}_{k},{\hat{h}}_{k},{\hat{c}}_{k},{\hat{s}}_{k})\right\}}_{k=1}^{\hat{n}}$, where ${\hat{s}}_{k}\in [0,1]$ denotes the confidence score, $\hat{n}$ is the number of predicted objects, and $\hat{n}$ is further used as the lesion counting result for the image. The parameters Θ are optimized by minimizing the detection loss $L_{det}(\text{Θ})$, such that the predicted boxes approximate the ground-truth annotations B as closely as possible in terms of localization accuracy and classification correctness.

### 3.2 Overall model architecture

**Figure 1:**
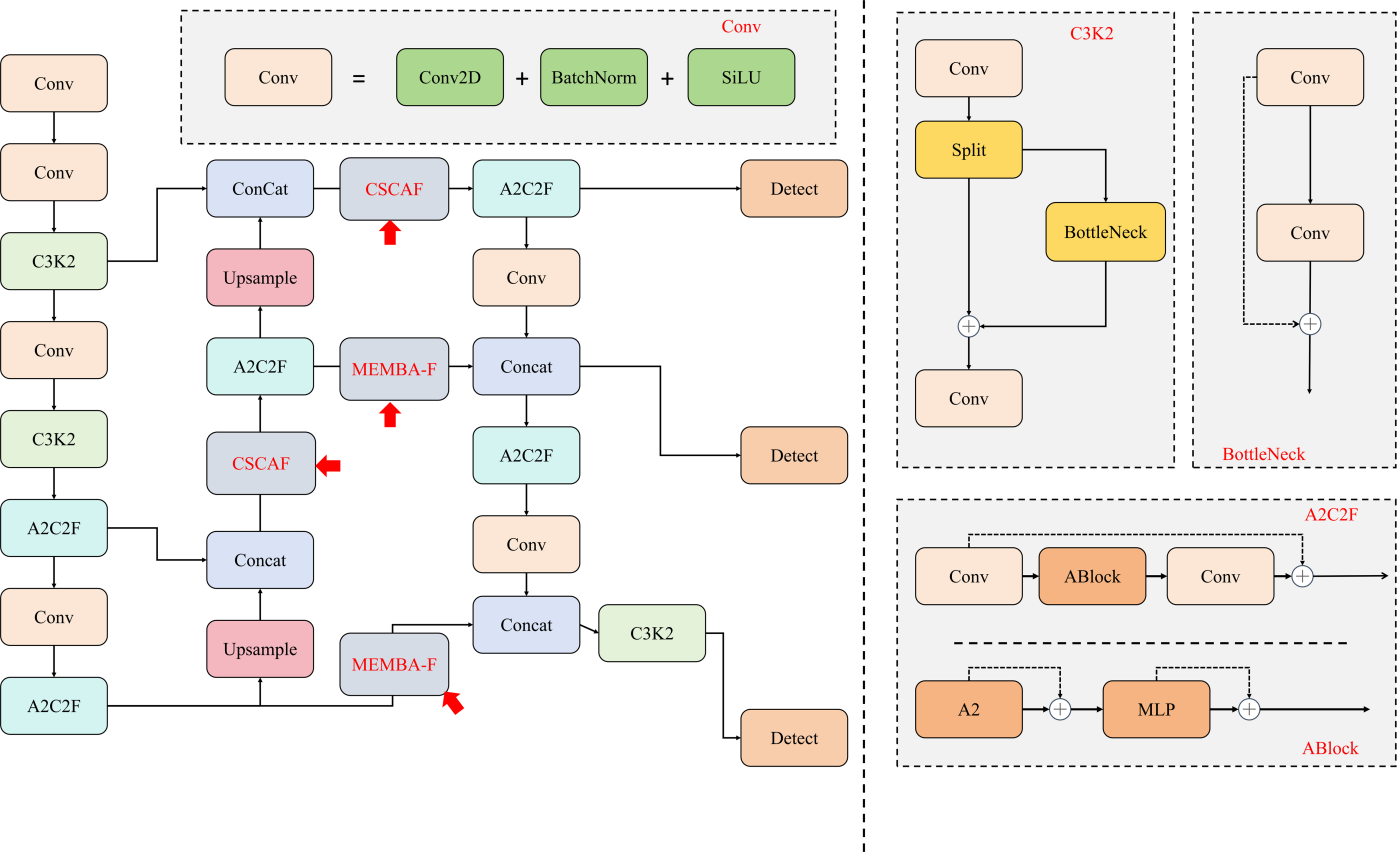
Based on the overall detection framework of YOLOv10, MEMBA-F is introduced in the neck feature fusion path for multi-scale feature enhancement, and CSCAF is introduced to achieve cross-scale context-aware fusion to improve the representation and alignment of small lesion targets. The right side shows the structural diagram of the key basic modules, including Conv, C3K2, BottleNeck, and A2C2F and its internal ABlock, which are used to illustrate the backbone and fusion unit design of the network in this paper.

The framework of the paper is based on the YOLOv10 framework and the conventional Backbone, Neck, and Head architecture for the end to end real time object detection and counting. Though the efficiency of one-stage object detectors is retained, the YOLOv10 architecture improves the end to end usability for deployment by using a more consistent prediction and matching approach to filter out redundant candidates, thus achieving a desirable accuracy-speed trade-off without the need for complex post-processing. Compared with newer YOLO variants such as YOLOv12, YOLOv10 has a smaller parameter scale and a more lightweight architecture, making it more suitable as the base detector for potential edge-device deployment scenarios. Based on the backbone, two new modules, MEMBA-F and CSCAF, are proposed to be added at critical locations in the feature flow to address the characteristics of leaf lesion detection, such as the size of the objects, the large number of instances, the fragmented texture, and the significant cross-scale variation. It should be emphasized that the proposed framework is not intended as a simple accumulation of generic YOLO components, but as a task-oriented redesign of the neck representation process according to the weak-texture, small-scale, high-density, and cross-scale characteristics of plant disease and pest targets. The first module, MEMBA-F, is for the multi-scale feature enhancement to enhance the discriminability of the weak lesions, and the second module, CSCAF, is for the cross-scale context-aware fusion to address the semantic gap between the cross-scale levels and improve the cross-scale feature alignment Liu et al. (2026). In this way, detection and counting are jointly supported by improving lesion-level feature visibility and cross-scale localization consistency, so that the final counting result is derived from more reliable instance-level predictions rather than from an independent *post hoc* statistical operation. The architecture of the proposed model is shown in **Figure 1**.

In terms of structural implementation, the Backbone extracts hierarchical features at different resolutions, {**C**_3_, **C**_4_, **C**_5_}, and the Neck constructs multi scale pyramid features via upsampling and concatenation, where CSCAF is injected during fusion to obtain stronger cross scale contextual modeling capability. This process can be summarized by the following mapping. As shown in Equation 1.

(1) $$\left\{{\text{P}}_{3},{\text{P}}_{4},{\text{P}}_{5}\}=N(M({\text{C}}_{3},{\text{C}}_{4},{\text{C}}_{5})\right)$$

where M(·) denotes the multi scale feature enhancement operator driven by MEMBA-F, N(·) denotes the feature pyramid construction operator that performs cross level fusion and injects CSCAF, and P*_l_*is the level *l* fused feature used for prediction in the detection head. Subsequently, the detection head outputs class confidence scores and bounding box predictions on {P_3_,P_4_,P_5_}, forming the prediction set $\hat{B}={\left\{({\hat{b}}_{k},{\hat{s}}_{k},{\hat{c}}_{k})\right\}}_{k=1}^{K}$, and the number of high confidence predictions is used as the lesion counting result to realize an integrated detection and counting output. The counting process can be expressed as follows. As shown in Equation 2.

(2) $$\hat{n}=\sum_{k=1}^{K}I\left({\hat{s}}_{k}\geq \tau \right)$$

where ${\hat{s}}_{k}\in [0,1]$ is the confidence score of the *k* th prediction, *τ* is the threshold, I(·) is the indicator function, and $\hat{n}$ is the final predicted number of lesion instances.

### 3.3 Multi-scale efficient Mamba block for feature enhancement

**Figure 2:**
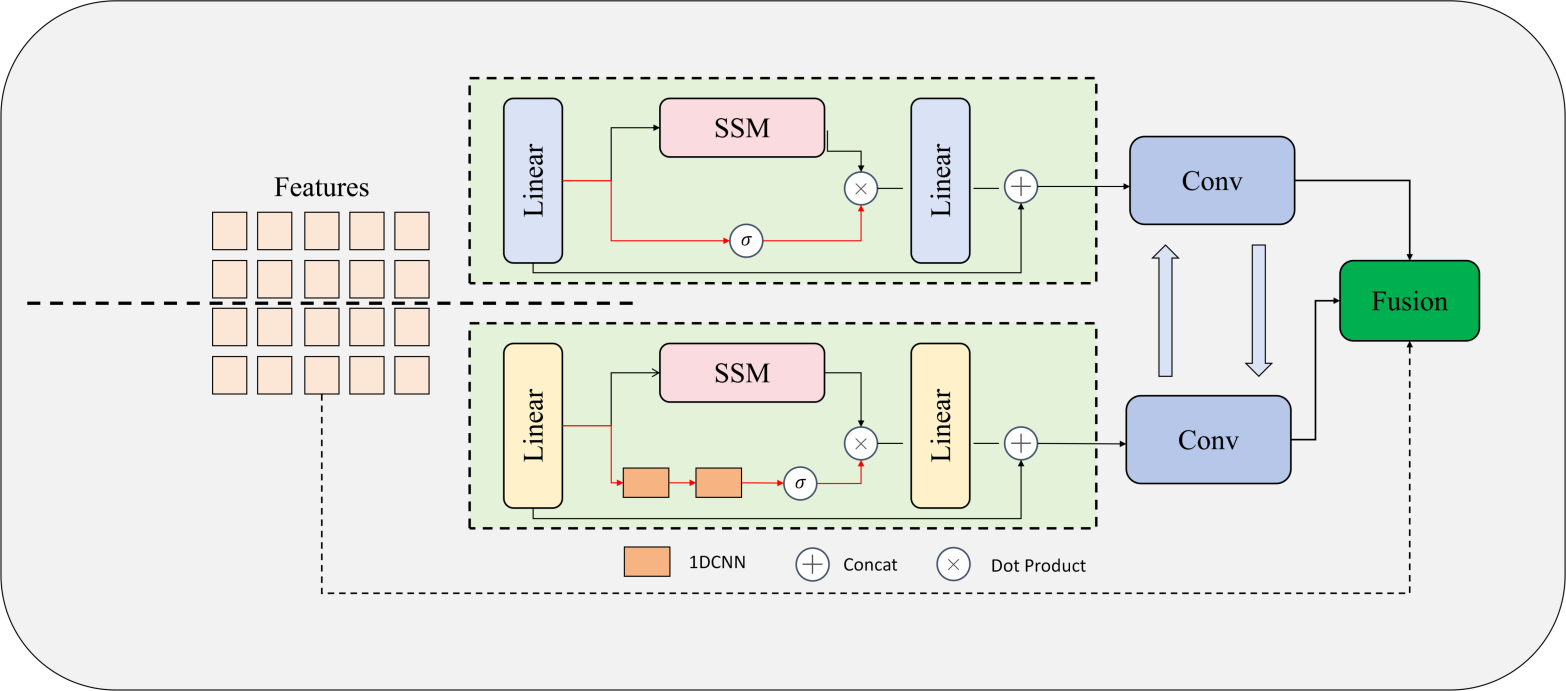
This image illustrates the structure of the multi-scale efficient Mamba feature enhancement module MEMBA-F. The upper branch drives the SSM to achieve global dependency modeling through linear projection and gating mechanisms. The lower branch introduces a lightweight 1D convolution before entering the SSM to enhance local continuous texture and fine-grained lesion patterns. The outputs of the two branches are convolved, aligned, and adaptively fused, then reinjected into the input features as residuals. This achieves both improved cross-scale semantic consistency and small lesion response capability with low overhead.

To better highlight leaf lesion features from cluttered field backgrounds, this paper proposes an efficient Mamba feature enhancement module, namely MEMBA-F, which operates at multiple scales. The main concept of MEMBA-F is that it preserves the ability of convolution to handle local textures, as well as the ability of state-space models to handle long-range dependencies. Different from a generic combination of convolution and Mamba, MEMBA-F is designed for plant disease and pest detection by jointly considering weak lesion texture, small target responses, dense spatial co-occurrence, and complex field background interference. As shown in **Figure 2**, the structure of MEMBA-F can be described as follows.

Let the input feature be $\text{X}\in {\mathbb{R}}^{C\times H\times W}$, where *C* is the number of channels and *H* and *W* denote the height and width of the feature map. To meet the sequence modeling requirement of SSMs, the two dimensional feature is first rearranged into a sequence representation $\text{Z}\in {\mathbb{R}}^{L\times C}$ in a spatial order, where L=H·W is the sequence length and the rearrangement operator is denoted by R(·), yielding: As shown in Equation 3.

(3) $$\text{Z}=R(\text{X}),\text{ Z}=[{\text{z}}_{1};{\text{z}}_{2};\ldots ;{\text{z}}_{L}],\;{\text{z}}_{t}\in {\mathbb{R}}^{C}.$$

Here, z*_t_* denotes the channel vector corresponding to the *t* th spatial position, which enables subsequent components to explicitly capture contextual correlations across positions.

Within a single scale branch, MEMBA-F constructs a controllable information flow using linear projections and a gating mechanism, and feeds it into an SSM to realize global dependency modeling with linear complexity. Specifically, given Z, a pair of linear projections are applied to obtain a content component and a gating component: As shown in Equation 4.

(4) $$\text{U}={\text{ZW}}_{u}+{\text{b}}_{u},\text{ G}=\sigma ({\text{ZW}}_{g}+{\text{b}}_{g}),$$

where ${\text{W}}_{u},{\text{W}}_{g}\in {\mathbb{R}}^{C\times d}$ are learnable weight matrices, ${\text{b}}_{u},{\text{b}}_{g}\in {\mathbb{R}}^{d}$ are biases, d is the hidden dimension, σ(·) is the Sigmoid function, $\text{U}\in {\mathbb{R}}^{L\times d}$ is the content feature, and $\text{G}\in {\mathbb{R}}^{L\times d}$ is the gating weight that suppresses noisy responses and highlights lesion related patterns. This gate is introduced to reduce responses from leaf veins, shadows, soil, and other background textures that are visually similar to pest or lesion regions. The gated feature is then fed into the SSM to obtain a globally enhanced representation $\text{Y}\in {\mathbb{R}}^{L\times d}$: As shown in Equation 5.

(5) Y=SSM(U⊙G),

where ⊙ denotes element wise multiplication and SSM(·) denotes a selective state space operator following the Mamba paradigm. By using input dependent dynamic parameters, it propagates key information selectively, making it more suitable for extracting long range discriminative cues under conditions with complex leaf textures and strong background interference.

To accommodate lesions at different scales, such as tiny spot like patterns, patch level expansions, and multi region dispersion, MEMBA-F introduces a two branch multi scale interaction structure. The upper branch focuses on efficient global dependency modeling, while the lower branch injects a lightweight one dimensional convolution before the SSM to strengthen local continuity patterns, forming a complementary combination of local priors and global propagation. This design enables the module to preserve fine lesion boundaries for small targets while modeling the spatial co-occurrence of multiple disease or pest instances on the same leaf. Let the one dimensional convolution operator in the lower branch be Conv1D(·;k) with kernel size k, then the lower branch can be written as: As shown in Equation 6.

(6) $$\tilde{\text{U}}=\text{Conv}1\text{D}(\text{U};k),\;\tilde{\text{Y}}=\text{SSM}(\tilde{\text{U}}⊙\text{G}),$$

where $\tilde{\text{U}}\in {\mathbb{R}}^{L\times d}$ is the locally mixed sequence representation and $\tilde{\text{Y}}\in {\mathbb{R}}^{L\times d}$ is its corresponding globally enhanced output. This design equips the module with both sensitivity to local lesion boundaries and texture changes, and the capability to model cross region consistency, such as the co-occurrence relationship of multiple lesions on the same leaf.

Finally, MEMBA-F performs explicit fusion at the scale level and restores the result to the two dimensional feature domain via convolutional projection, enabling seamless integration with the YOLOv10 neck feature flow. Let the fusion operator be F(·) and the inverse rearrangement operator be $R^{-1}(\cdot )$, and let a 1×1 convolution ϕ(·) be used for channel alignment. The module output ${\text{X}}^{\prime }\in {\mathbb{R}}^{C\times H\times W}$ is defined as: As shown in Equation 7.

(7) $${\text{X}}^{\prime }=\phi (R^{-1}(F([\text{Y}\|\tilde{\text{Y}}])))+\text{X},$$

where [· ‖ ·] denotes feature concatenation, F(·) can be instantiated as a position wise linear fusion or an attention weighted fusion to adaptively balance the contributions of the two branches, and the residual term +X preserves the original local details and improves training stability. With these designs, MEMBA-F achieves local texture strengthening and global context enhancement for multi scale lesions under controlled computational overhead, providing a more discriminative feature basis for subsequent cross scale fusion and detection head prediction.

### 3.4 Cross-scale context-aware fusion

**Figure 3:**
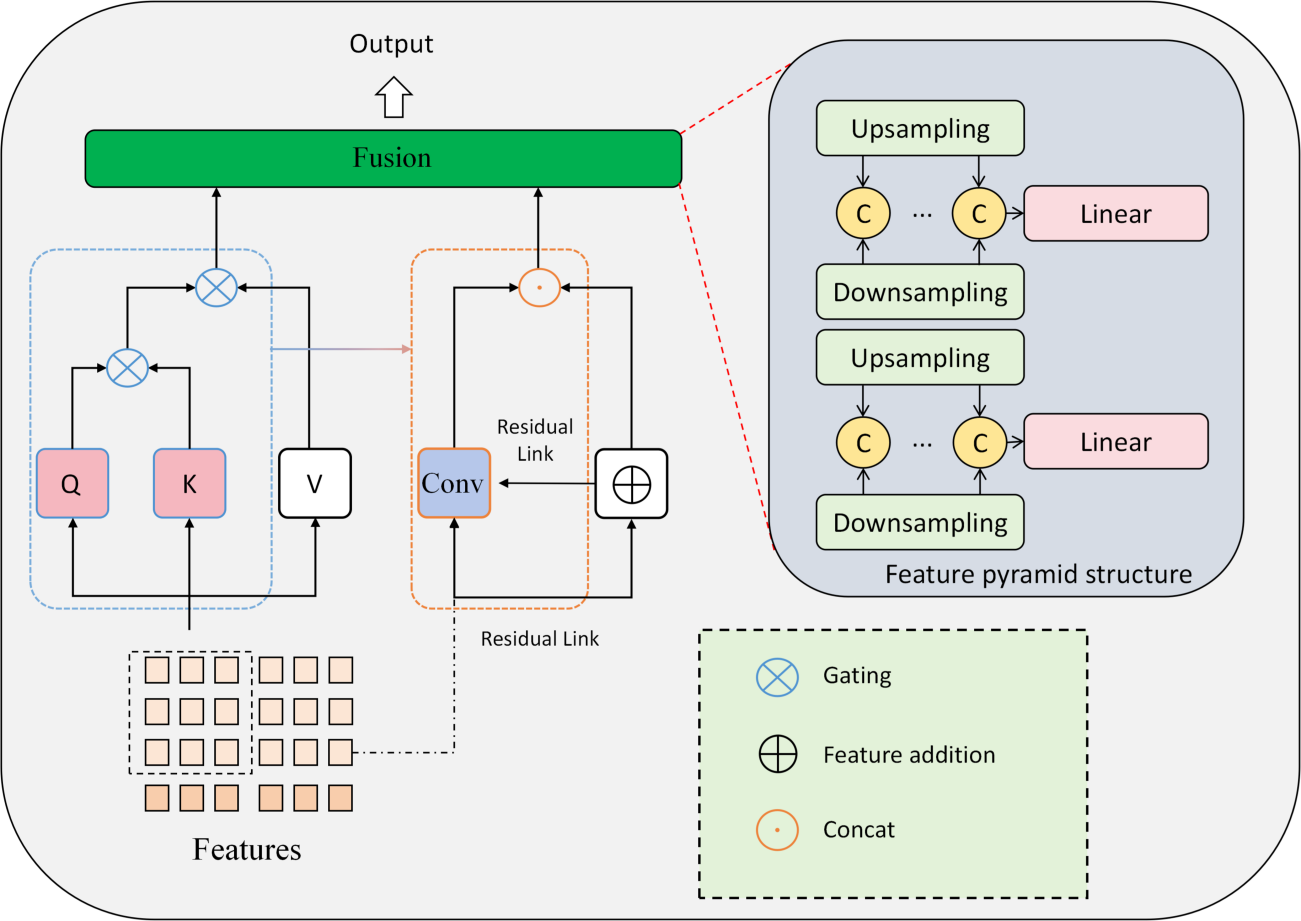
This image illustrates the structure of the cross-scale context-aware fusion module CSCAF. Features from different pyramid levels are first spatially and channel aligned, and the target scale feature is used as the query to aggregate complementary semantic and fine-grained information from other scales. A gating unit is further introduced to selectively retain lesion or pest-related cross-scale responses while suppressing irrelevant background textures, and the selected context is reinjected into the target feature through a residual connection.

To keep consistency with the YOLOv10 based Backbone, Neck, and Head architecture and to fully exploit the multi scale representations enhanced by MEMBA-F, we introduce a cross scale context aware fusion module, termed CSCAF, at the cross level aggregation positions in the Neck. Targeting the key challenges of leaf lesion detection, including many instances of small lesions, significant cross scale morphological variations, and strong background texture interference in field scenes, CSCAF explicitly constructs a fusion mechanism inside the feature pyramid that combines cross scale context aggregation, gating based selection, and residual reinjection, thereby alleviating alignment bias caused by insufficient semantics in shallow features and missing details in deep features. Different from a generic cross-scale attention module, CSCAF is designed to address the inconsistency between small target localization cues and high-level semantic cues in plant disease and pest scenes, where lesions and pests are often weakly textured, densely distributed, and easily confused with leaf veins or background noise. Let the multi scale feature set from the Neck be {F_3_, F_4_, F_5_}, where ${\text{F}}_{l}$ ∈ ℝ *^Cl^*^×^*^Hl^*^×^*^Wl^* corresponds to three feature levels from high resolution to low resolution, and *H_l_*, *W_l_*, and *C_l_*denote the spatial size and channel dimension. CSCAF anchors on a target scale *s* ∈{3,4,5} to construct cross scale context and outputs a fused feature ${\text{Y}}_{\text{s}}\in {\mathbb{R}}^{C\times H_{s}\times W_{s}}$ for the subsequent detection head. Finally, a module architecture diagram of Cross-Scale Context-Aware Fusion is also provided, as shown in **Figure 3**.

CSCAF first aligns non target scale features by upsampling or downsampling and unifies the channel dimension to enable cross scale interaction. The alignment operator is denoted by $T_{l\to s}(\cdot )$, which consists of Resize(·) and a 1×1 linear projection ϕ(·), producing the aligned feature ${\text{X}}_{l}^{\left(s\right)}\in {\mathbb{R}}^{C\times H_{s}\times W_{s}}$ as: As shown in Equation 8.

(8) $${\text{X}}_{l}^{\left(s\right)}=T_{l\to s}({\text{F}}_{l})=\phi (\text{Resize}({\text{F}}_{l};H_{s},W_{s})),\;l\in \{3,4,5\}.$$

Here, $\text{Resize}({\text{F}}_{l};H_{s},W_{s})$ resamples ${\text{F}}_{l}$ to $\left(H_{s},W_{s}\right)$, and ϕ(·) maps the channels from $C_{l}$ to a unified C. This alignment corresponds to the upsampling and downsampling pyramid links in the right part of the figure, making different scale features comparable on the same spatial grid and providing a unified computation domain for subsequent cross scale context extraction.

After alignment, CSCAF takes the target scale feature $X_{s}^{\left(s\right)}$ as the Query and uses the remaining scales ${\left\{{\text{X}}_{l}^{\left(s\right)}\right\}}_{l\neq s}$ as context sources to construct cross scale context aware Q, K, and V interaction for absorbing complementary information. This target-scale anchored design preserves the spatial positions of small disease or pest candidates while introducing complementary context from other scales, which is important for reducing missed detections and localization shifts in dense field scenes. We flatten the two dimensional features into sequences with length $N=H_{s}W_{s}$ and obtain embeddings ${\text{Q}}_{s}$, ${\text{K}}_{l}$, and ${\text{V}}_{l}\in {\mathbb{R}}^{N\times d}$ via 1×1 convolution based projections: As shown in Equation 9.

(9) $${\text{Q}}_{s}=\psi_{q}(\text{Flat}({\text{X}}_{s}^{\left(s\right)})),\;{\text{K}}_{l}=\psi_{k}(\text{Flat}({\text{X}}_{l}^{\left(s\right)})),\;{\text{V}}_{l}=\psi_{v}(\text{Flat}({\text{X}}_{l}^{\left(s\right)})).$$

Here, Flat(·) flattens features by spatial locations, d is the embedding dimension, and $\psi_{q}$, $\psi_{k}$, and $\psi_{v}$ are learnable linear mappings. A scaled dot product correlation is then used to generate cross scale attention weights and perform context aggregation, producing the context representation injected from scale l to scale s, denoted by ${\text{C}}_{l\to s}\in {\mathbb{R}}^{N\times d}$: As shown in Equation 10.

(10) $${\text{A}}_{l\to s}=\text{Softmax}\;(\frac{{\text{Q}}_{s}{\text{K}}_{l}^{⊤}}{\sqrt{d}}),\;{\text{C}}_{l\to s}={\text{A}}_{l\to s}{\text{V}}_{l},\;l\neq s.$$

Here, ${\text{A}}_{l\to s}\in {\mathbb{R}}^{N\times N}$ measures the correlation between each target scale location and each context scale location, enabling selective alignment and injection of strong semantics from deeper levels or fine grained textures from shallower levels according to relevance to the current lesion candidate position. This design matches the assumption in our task that lesion morphology varies across scales while consistent cues exist for the same lesion region at multiple scales.

Since field leaf backgrounds often contain strong texture interference, direct cross scale aggregation may introduce irrelevant information and lead to false detections. CSCAF further performs gating based selection, shown as a Gating unit in the figure, and stabilizes reinjection via a residual link. Specifically, we combine aggregated contexts from all sources and learn a position adaptive gate ${\text{g}}_{s}\in {\mathbb{R}}^{N\times 1}$, while applying learnable weighting to balance the contributions from different scales: As shown in Equation 11.

(11) $${\text{C}}_{s}=\underset{l\neq s}{\sum }\alpha_{l\to s}\;{\text{P}}_{l}\;{\text{C}}_{l\to s},\;{\text{g}}_{s}=\sigma \;({\text{W}}_{g}[{\text{C}}_{3\to s}\|{\text{C}}_{4\to s}\|{\text{C}}_{5\to s}]+{\text{b}}_{g}),$$

where $\alpha_{l\to s}\in \mathbb{R}$ is a scale weight that can be learnable or generated from global pooling, ${\text{P}}_{l}\in {\mathbb{R}}^{d\times d}$ is a source specific subspace projection matrix, [·‖·] denotes concatenation, ${\text{W}}_{g}\in {\mathbb{R}}^{(md)\times 1}$ and ${\text{b}}_{g}\in \mathbb{R}$ are gating parameters, *m* is the number of context sources involved in fusion, and *σ*(·) is the Sigmoid function. The gate serves as a lesion-aware selection mechanism, allowing the model to suppress cross-scale responses from leaf texture, illumination variation, and occlusion while retaining discriminative cues associated with true disease or pest instances. The gated context ${\tilde{\text{C}}}_{s}={\text{g}}_{s}⊙{\text{C}}_{s}$ is then restored to the two dimensional layout, channel aligned by a convolution, and reinjected with a residual formulation to obtain the output feature ${\text{Y}}_{s}$: As shown in Equation 12.

(12) $${\text{Y}}_{s}={\text{X}}_{s}^{\left(s\right)}+\eta_{s}\cdot \rho (\text{Unflat}({\tilde{\text{C}}}_{s})),$$

where ⊙ denotes element wise multiplication, Unflat(·) reshapes ${\mathbb{R}}^{N\times d}$ back to ${\mathbb{R}}^{d\times H_{s}\times W_{s}}$, *ρ*(·) is a 1 × 1 convolution that maps the channel dimension from *d* back to *C*, and *η_s_*is a scale dependent injection coefficient. This gating and residual design complements MEMBA-F, 303 where MEMBA-F enhances lesion textures and long range consistency within a single scale, while CSCAF suppresses the propagation of background noise during cross scale interaction and strengthens consistent alignment of multi scale evidence, thereby providing more robust fused features for the YOLOv10 detection head to support lesion instance detection and counting.

### 3.5 Training objective and loss function

The proposed model follows YOLOv10 as the training paradigm, and optimizes MEMBA-F and CSCAF together with the Neck in an end to end manner, so that multi scale lesion features can form discriminative representations that are more sensitive to small targets and weak texture during training. Let the network parameters be Θ. Given an input image *I*, the Backbone, Neck, and Head produce predictions at three scales, denoted by $\hat{P}={\left\{{\hat{P}}_{s}\right\}}_{s\in \{3,4,5\}}$. A matching set $M={\left\{(a_{k},g_{k})\right\}}_{k=1}^{K}$ is obtained using the same positive and negative sample assignment strategy as YOLOv10, where *a_k_*denotes the prediction unit at the anchor position of the *k* th positive sample and $g_{k}=(b_{k},c_{k})$ is the matched ground truth annotation consisting of a bounding box *b_k_*and a class label *c_k_*. The overall training objective is composed of an IoU based bounding 317 box regression loss, a distributional bounding box refinement loss, and a classification loss: As shown in Equation 13.

(13) $$L(\text{Θ})=\lambda_{\text{iou}}L_{\text{iou}}+\lambda_{\text{dfl}}L_{\text{dfl}}+\lambda_{\text{cls}}L_{\text{cls}}$$

where *λ*_iou_, *λ*_dfl_, and *λ*_cls_ are the weights for the three loss terms, $L_{\text{iou}}$ constrains geometric consistency between predicted and ground truth boxes, $L_{\text{dfl}}$ improves fine grained boundary regression accuracy, and $L_{\text{cls}}$ optimizes class discriminability.

For bounding box regression, let ${\hat{b}}_{k}$ be the predicted box and *b_k_*be the ground truth box for the *k* th positive sample, then the IoU style regression term can be written as: As shown in Equation 14.

(14) $$L_{\text{iou}}=\frac{1}{K}\sum_{k=1}^{K}\left(1-\text{IoU}\left({\hat{b}}_{k},b_{k}\right)\right)$$

where IoU(·,·) is the intersection over union function and *K* is the number of positive samples. To achieve sub pixel level delineation of small lesion boundaries, YOLOv10 adopts the distributional regression idea that is consistent with modern YOLO families, discretizing boundary distances into *M* bins and predicting a probability distribution Let the discrete distributions along the left, upper, right, and lower directions for the k th sample be ${\hat{\text{p}}}_{k}^{\left(t\right)}\in {\mathbb{R}}^{M}$, and let the corresponding ground truth soft labels be ${\text{q}}_{k}^{\left(t\right)}\in {\mathbb{R}}^{M}$, where t∈{l,u,r,d}. The distributional refinement term is defined as: As shown in Equation 15.

(15) $$L_{\text{dfl}}=\frac{1}{4K}\sum_{k=1}^{K}\sum_{t\in \left\{l,u,r,d\right\}}\text{CE}\left({\text{q}}_{k}^{\left(t\right)},{\hat{\text{p}}}_{k}^{\left(t\right)}\right)$$

where CE(·,·) denotes cross entropy and *M* is the number of discrete bins. This term can significantly improve the stability and accuracy of boundary localization, complementing the small target texture cues enhanced by MEMBA-F. The classification term adopts a binary cross entropy form. Let ${\hat{\text{s}}}_{k}\in {\left[0,1\right]}^{C}$ denote the predicted class score vector for the k th positive sample and let ${\text{y}}_{k}\in {\left\{0,1\right\}}^{C}$ be the ground truth one hot label, where C is the number of lesion categories, then: As shown in Equation 16.

(16) $$L_{\text{cls}}=\frac{1}{K}\sum_{k=1}^{K}\sum_{c=1}^{C}\text{BCE}\left(y_{k,c},{\hat{s}}_{k,c}\right)$$

where BCE(·,·) denotes binary cross entropy and $y_{k,c}$ and ${\hat{s}}_{k,c}$ denote the ground truth and predicted components for class *c*, respectively. Since CSCAF explicitly performs cross scale context gated fusion in the Neck, the three supervision terms jointly drive improvements in cross scale alignment and fine grained localization, which better matches the requirements of multi instance lesion counting scenarios for high recall on small targets and low false positives. The corresponding training procedure is summarized in **Algorithm 1**.

Algorithm 1

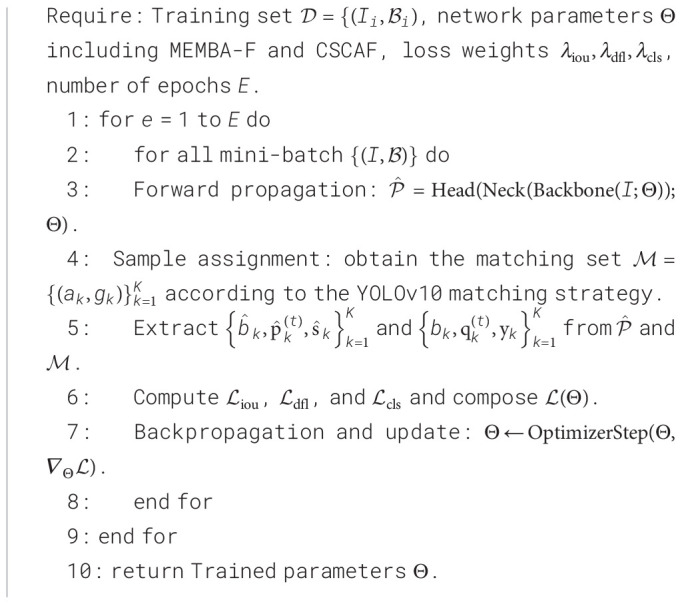

## 4 Datasets and evaluation metrics

### 4.1 Dataset

#### 4.1.1 Dataset 1 (PlantDoc)

This study adopts the publicly available plant disease object detection dataset PlantDoc as the first experimental dataset. Proposed by Singh et al., this dataset targets plant disease visual detection in natural field environments. The image acquisition conditions include real farmland backgrounds, natural illumination variations, leaf occlusion and overlap, as well as complex texture interference. Compared with standardized laboratory settings, the detection difficulty is significantly increased. PlantDoc contains 2,569 images, covering 13 plant species and 30 categories (including healthy and different disease types), with a total of 8,851 annotated bounding boxes. On average, each image contains approximately 3.44 disease instances, exhibiting a clear multi-instance and small-object distribution. The dataset provides object-level annotations, making it suitable for training and evaluating single-stage detectors under the YOLO framework. Meanwhile, its complex structure across species and disease categories offers a solid testbed for validating the model’s cross-scale representation capability and robustness against background interference. To ensure training stability and computational efficiency, a preprocessed version with a unified resolution of 416×416 is used in our experiments. The overall statistics of the dataset are summarized in **Table 1**.

**Table 1:** Statistics of the PlantDoc dataset.

Item	Value
Number of images	2,569
Number of plant species	13
Number of classes (including healthy)	30
Total number of bounding boxes	8,851
Average instances per image	3.44
Image resolution	416×416
Task type	Object detection

#### 4.1.2 Dataset 2

**Figure 4:**
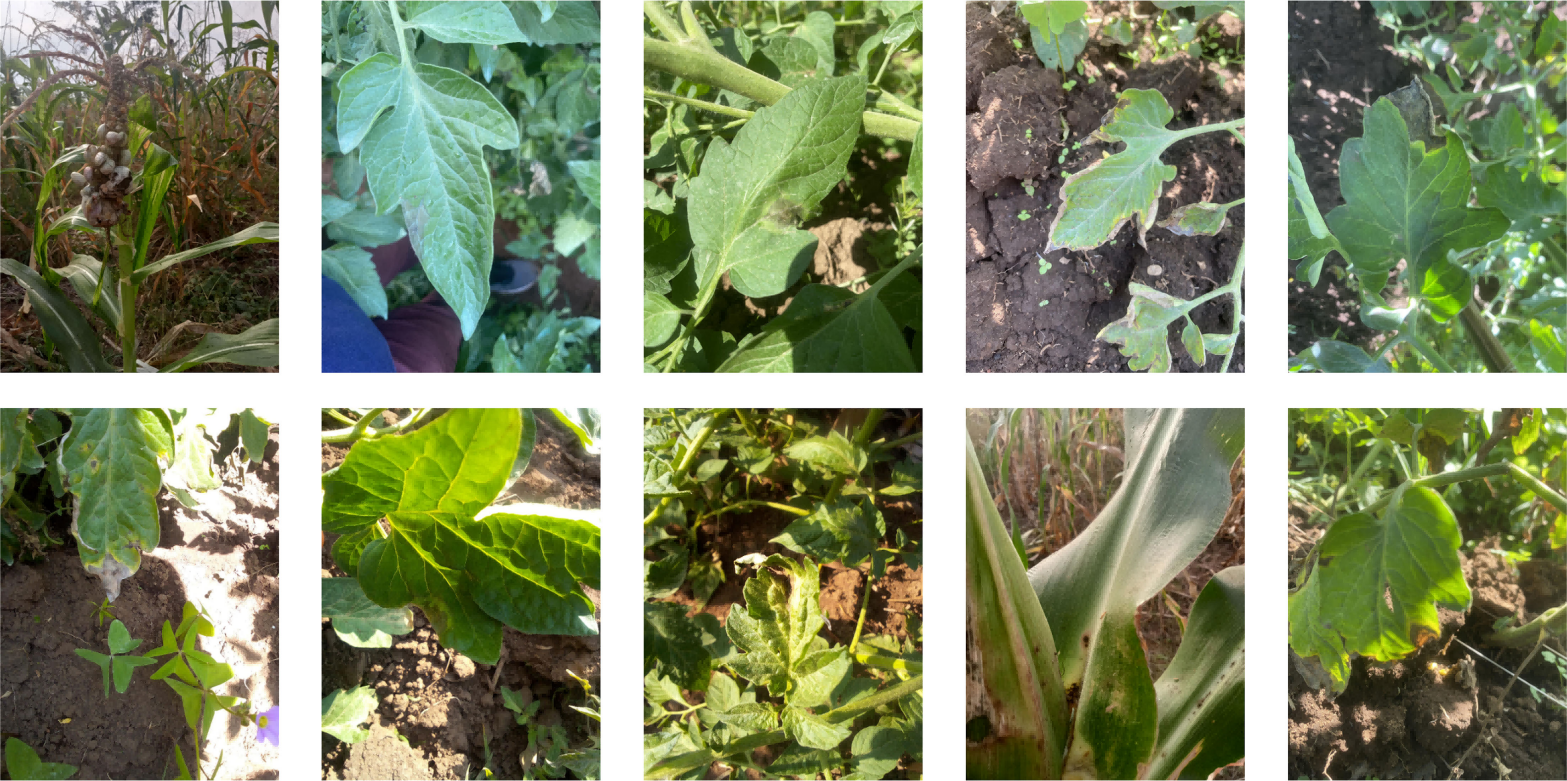
Example of dataset 2.

In order to test the model’s ability to generalize to more challenging, realistic agricultural situations involving multiple crops and different types of diseases, we will utilize a second public object detection dataset for plant diseases from Kaggle. This dataset is ready to be used for the YOLO object detection model. It is split into a set of training, validation, and test images. The dataset contains 27 different classes of images across three of the most important crops: cassava, corn, and tomato. The images contain a variety of different diseases, as well as healthy leaves. The images are taken from a variety of different settings in the wild, where there is strong variation in lighting, a cluttered background, leaf occlusions, and the occurrence of multiple instances of the object of interest. The difference among the object categories is obvious in terms of texture and color. Additionally, there are small lesions or localized symptoms, which requires strong feature modeling across different scales. This dataset will be used to test the strength of the MEMBA-F and CSCAF modules in a challenging, multi-class object detection problem. This will add to the credibility of the results. An example of this object detection dataset is shown in **Figure 4**.

#### 4.1.3 Dataset 3

**Figure 5:**
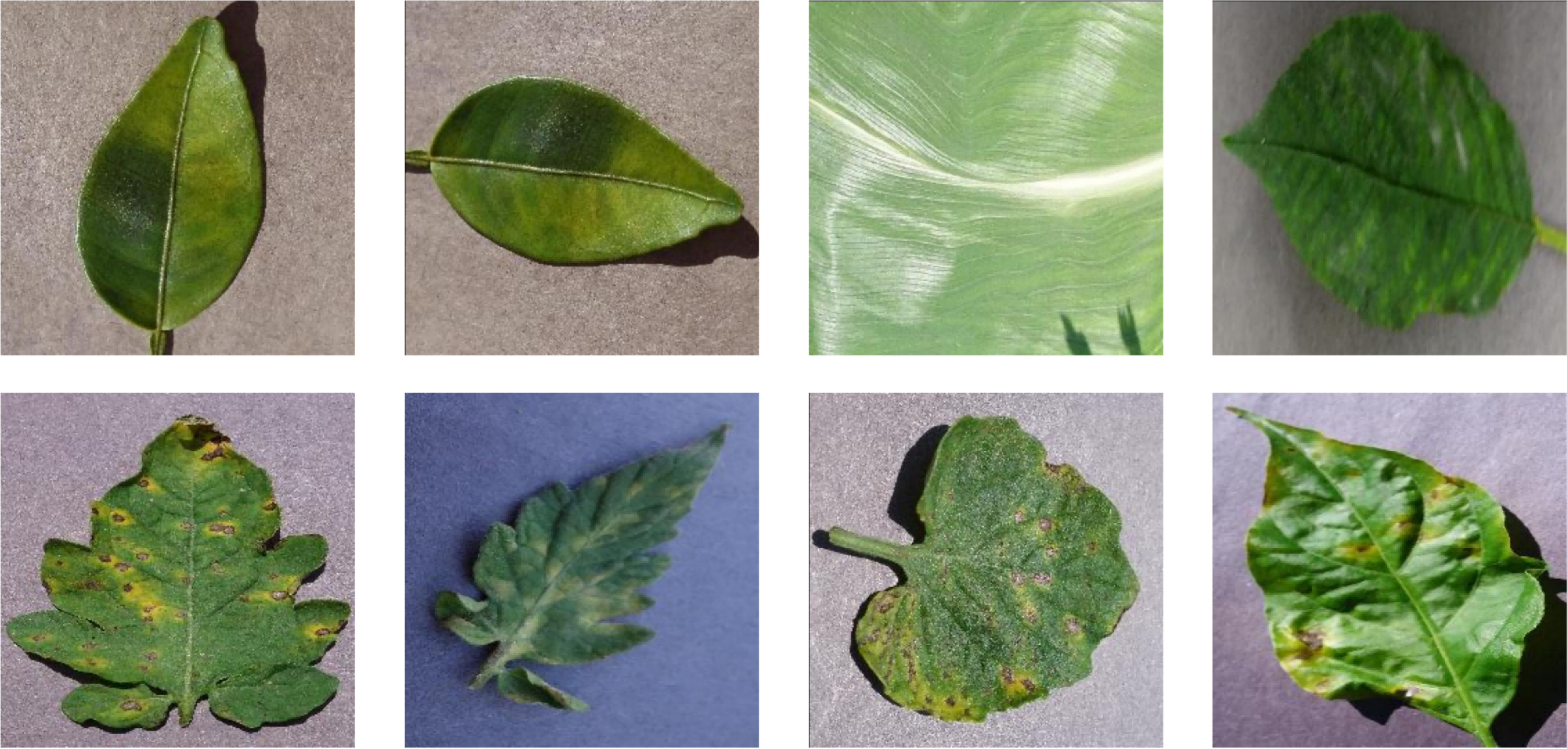
Example of dataset 3.

To further expand the limits of scale and evaluate how well this model can perform in large, real-world agricultural settings, this study also includes a third public dataset for plant disease object detection. This dataset contains 29,012 images, with leaf instances being labeled as bounding boxes in YOLOv8 format. These are all uniformly resized to a resolution of 640×640. These are pulled from various sources, allowing for a variety of leaf shapes and settings with cluttered backgrounds, as can be expected from real-world farms. There are also notable changes in scale, as well as dense instances of leaves and various viewpoints. As part of preprocessing, automatic orientation correction and normalization of sizes are implemented. A 90-degree rotation augmentation also provides various viewpoints of each sample, increasing robustness to spatial orientations. Although this dataset may seem similar to the first two, it must be noted that this dataset is more extensive and varied in sample distribution, allowing for a more complete evaluation of how well this model can perform in complex scenarios. Sample images from this dataset are shown in **Figure 5**.

#### 4.1.4 Dataset statistical analysis

To improve the transparency of the dataset characteristics, a statistical analysis of object size distribution was conducted based on the annotated bounding boxes. For each annotated object, the normalized bounding-box area ratio was calculated as $r_{i}=w_{i}h_{i}/(W_{i}H_{i})$, where *w_i_*and *h_i_*denote the width and height of the bounding box, and *W_i_*and *H_i_*denote the width and height of the corresponding image. Based on this normalized area ratio, objects were divided into three relative size groups: small objects with *r_i_ <* 0.05, medium objects with 0.05 ≤ *r_i_ <* 0.15, and large objects with *r_i_*≥ 0.15. This relative definition avoids the influence of different image resolutions and provides a unified criterion for comparing the object-size distributions of Dataset1, Dataset2, and Dataset3. The statistical results are summarized in **Table 2**.

**Table 2:** Object size distribution statistics of Dataset1–Dataset3.

Dataset	Small (%)	Medium (%)	Large (%)	Total (%)
Dataset 1	62.06	22.66	15.27	100.00
Dataset 2	24.97	34.69	40.35	100.00
Dataset 3	11.09	12.44	76.47	100.00

### 4.2 Evaluation metrics

To evaluate the model’s effectiveness in handling plant disease object detection, this study utilizes Precision, Recall, mAP@50, and mAP@50–95 as primary metrics. These metrics enable a quantitative evaluation of the model from various aspects, including its precision, as well as its ability to recall positively classified objects.

Precision measures the proportion of true positives among all predicted positives, reflecting the accuracy of detection results. It is defined as: As shown in Equation 17.

(17) $$Precision=\frac{TP}{TP+FP}$$

where *TP* denotes the number of correctly detected objects, and *FP* denotes the number of false detections. A higher Precision indicates a lower false positive rate.

Recall measures the proportion of ground-truth objects that are correctly detected, reflecting the model’s coverage capability. It is defined as: As shown in Equation 18.

(18) $$Recall=\frac{TP}{TP+FN}$$

where *FN* denotes the number of ground-truth objects that are missed by the detector. A higher Recall indicates a lower miss rate.

mAP@50 denotes the mean of Average Precision (AP) over all classes under an IoU threshold of 0.5. First, the AP for a single class at an IoU threshold of 0.5 is defined as: As shown in Equation 19.

(19) $$AP_{50}=\int_{0}^{1}Precision(Recall)\;dRecall$$

Then, the mean over all classes is computed as: As shown in Equation 20.

(20) $$mAP@50=\frac{1}{C}\sum_{c=1}^{C}AP_{50}^{\left(c\right)}$$

where *C* denotes the total number of classes. This metric mainly reflects the overall detection capability under a moderate localization requirement.

mAP@50–95 is computed by averaging AP over multiple IoU thresholds ranging from 0.5 to 0.95 with a step size of 0.05. It is defined as: As shown in Equation 21.

(21) $$mAP@50-95=\frac{1}{10C}\sum_{t=0.5}^{0.95}\sum_{c=1}^{C}AP_{t}^{\left(c\right)}$$

where *t* denotes different IoU thresholds. This metric provides a more stringent evaluation of overall detection performance under varying localization requirements and is a comprehensive and representative standard widely used in object detection.

## 5 Experimental results and analysis

### 5.1 Experimental results compared with other models

To understand the efficacy of the proposed approach for plant disease and pest detection, we conduct a unified experiment using Dataset1. Here, the efficacy of the proposed approach is compared with other state-of-the-art models, including two-stage models such as Faster R-CNN and Mask R-CNN, and one-stage models such as the YOLO family, including YOLOv5, YOLOv7, YOLOv8, YOLOv11, and YOLOv12. Moreover, the efficacy of the proposed approach is compared with other models, including DETR, DAB-DETR, RT-DETR, and PD-TR, which are based on the transformer family. To understand the efficacy of the proposed approach, we consider several factors, including the accuracy of the models, localization and classification accuracy, and inference speed. For this purpose, we consider Precision, Recall, mAP@50, mAP@50-95, and inference time per image (ms) for evaluating the efficacy of the proposed approach and other models. Initially, the results for Dataset1 are presented in **Table 3**.

**Table 3:** Experimental results on Dataset1 compared with other models.

Method	Params (M)	Precision	Recall	mAP@50	mAP@50-95	Inference speed (ms)
Faster RCNN Ren et al. (2015)	41.8	0.441 ± 0.012	0.328 ± 0.015	0.493 ± 0.017	0.287 ± 0.014	42.8
Mask-RCNN He et al. (2017)	44.4	0.456 ± 0.014	0.335 ± 0.013	0.512 ± 0.018	0.296 ± 0.012	45.3
YOLOV5 Jocher et al. (2022)	21.2	0.489 ± 0.011	0.341 ± 0.014	0.526 ± 0.016	0.309 ± 0.013	37.1
YOLOV7 Wang et al. (2023a)	36.9	0.516 ± 0.013	0.356 ± 0.012	0.548 ± 0.015	0.324 ± 0.011	33.4
YOLOV8 Sohan et al. (2024)	25.9	0.532 ± 0.012	0.367 ± 0.013	0.561 ± 0.014	0.339 ± 0.012	30.2
YOLOV11 Hidayatullah et al. (2025)	20.1	0.548 ± 0.013	0.369 ± 0.011	0.569 ± 0.015	0.348 ± 0.013	28.7
DETR Carion et al. (2020)	41.4	0.503 ± 0.014	0.345 ± 0.015	0.542 ± 0.016	0.321 ± 0.014	49.6
YOLOV12 Tian et al. (2025)	20.2	0.559 ± 0.012	0.368 ± 0.014	0.574 ± 0.016	0.352 ± 0.013	27.1
RT-DETR Zhao et al. (2024)	42.0	0.538 ± 0.011	0.358 ± 0.012	0.556 ± 0.015	0.334 ± 0.012	26.8
DAB-DETR Liu et al. (2022)	44.0	0.527 ± 0.013	0.351 ± 0.014	0.551 ± 0.017	0.329 ± 0.013	40.7
PD-TR Wang et al. (2024)	47.0	0.556 ± 0.012	0.366 ± 0.013	0.567 ± 0.015	0.354 ± 0.012	23.2
Ours	17.2	**0.573 ± 0.011**	**0.372 ± 0.012**	**0.582 ± 0.014**	**0.365 ± 0.011**	27.4

Bold values indicates the optimal result.

As can be seen from **Table 3**, our method excels in detection results. Our method achieves the highest values of Precision, Recall, mAP@50, and mAP@50–95 among all compared methods. This indicates that our method can reduce false positives without compromising detection targets. In addition, our method achieves more consistent results with varying IoU thresholds. By comparing our method with two-stage detectors like Faster R-CNN and Mask R-CNN, our method achieves better accuracy metrics with higher efficiency in inference. By comparing our method with typical YOLO methods and some transformers, our method still achieves better accuracy metrics, and its efficiency in inference also falls within the range of real-time deployable detectors of the same category. The experimental results of datasets 2 and 3 are shown in **Tables 4**, **5**, respectively.

**Table 4:** Experimental results on Dataset2 compared with other models.

Method	Params (M)	Precision	Recall	mAP@50	mAP@50-95	Inference speed (ms)
Faster RCNN	41.8	0.542 ± 0.024	0.487 ± 0.031	0.534 ± 0.029	0.342 ± 0.022	42.8
Mask-RCNN	44.4	0.558 ± 0.028	0.496 ± 0.026	0.547 ± 0.031	0.354 ± 0.025	45.3
YOLOV5	21.2	0.583 ± 0.022	0.521 ± 0.027	0.561 ± 0.033	0.368 ± 0.024	37.1
YOLOV7	36.9	0.602 ± 0.026	0.533 ± 0.029	0.574 ± 0.032	0.379 ± 0.023	33.4
YOLOV8	25.9	0.628 ± 0.027	0.549 ± 0.030	0.588 ± 0.034	0.392 ± 0.026	30.2
YOLOV11	20.1	0.647 ± 0.025	0.561 ± 0.028	0.598 ± 0.037	0.403 ± 0.024	28.7
YOLOV12	20.2	0.662 ± 0.029	0.573 ± 0.031	0.602 ± 0.036	0.409 ± 0.027	27.1
DETR	41.4	0.571 ± 0.030	0.514 ± 0.034	0.553 ± 0.032	0.361 ± 0.028	49.6
RT-DETR	42.0	0.654 ± 0.023	0.587 ± 0.029	0.606 ± 0.035	0.412 ± 0.026	26.8
DAB-DETR	44.0	0.622 ± 0.028	0.554 ± 0.033	0.592 ± 0.038	0.398 ± 0.029	40.7
PD-TR	47.0	0.667 ± 0.031	0.611 ± 0.035	0.603 ± 0.039	0.416 ± 0.030	23.2
Ours	17.2	**0**.**691** ± **0**.**022**	**0**.**652** ± **0**.**028**	**0**.**614** ± **0**.**041**	**0**.**421** ± **0**.**031**	27.4

Bold values indicates the optimal result.

**Table 5:** Experimental results on Dataset3 compared with other models.

Method	Params (M)	Precision	Recall	mAP@50	mAP@50-95	Inference speed (ms)
Faster RCNN	41.8	0.361 ± 0.031	0.402 ± 0.027	0.421 ± 0.034	0.183 ± 0.022	42.8
Mask-RCNN	44.4	0.374 ± 0.035	0.418 ± 0.032	0.433 ± 0.038	0.191 ± 0.024	45.3
YOLOV5	21.2	0.392 ± 0.029	0.436 ± 0.033	0.451 ± 0.041	0.198 ± 0.026	37.1
YOLOV7	36.9	0.405 ± 0.034	0.448 ± 0.036	0.462 ± 0.043	0.203 ± 0.028	33.4
YOLOV8	25.9	0.416 ± 0.037	0.457 ± 0.034	0.471 ± 0.045	0.209 ± 0.027	30.2
YOLOV11	20.1	0.419 ± 0.033	0.468 ± 0.038	0.478 ± 0.047	0.213 ± 0.029	28.7
DETR	41.4	0.384 ± 0.036	0.429 ± 0.031	0.444 ± 0.039	0.195 ± 0.025	49.6
RT-DETR	42.0	0.423 ± 0.028	0.474 ± 0.037	0.486 ± 0.046	0.214 ± 0.030	26.8
DAB-DETR	44.0	0.412 ± 0.032	0.458 ± 0.035	0.473 ± 0.044	0.205 ± 0.027	40.7
PD-TR	47.0	0.421 ± 0.038	0.481 ± 0.040	0.489 ± 0.048	0.216 ± 0.031	23.2
YOLOV12	20.2	0.425 ± 0.041	0.489 ± 0.043	0.491 ± 0.049	**0.217 ± 0.033**	27.1
Ours	17.2	**0.427 ± 0.027**	**0.495 ± 0.039**	**0.492 ± 0.051**	**0.217 ± 0.032**	27.4

Bold values indicates the optimal result.

In the Dataset2 experiment, our method leads the chart in all four fundamental metrics: Precision, Recall, mAP@50, and mAP@50-95. This indicates a clear potential in eliminating false positives due to background interference while ensuring the detection targets, i.e., lesions and pests, are covered more comprehensively. This aligns well with the design philosophy: the use of MEMBA-F to enhance the features in the Neck region and the use of CSCAF to fuse the cross-scale contexts in the Neck region. The former increases the cross-scale responses in the detection of lesions when the textures are complex and the contrast is low. The latter aligns the high-level semantics and low-level details in the detection targets via cross-scale attention and fusion, hence eliminating the possibility of missed detection and localization shifts. With respect to the speed of the method, the experiment indicates that the method remains within the realm of real-time latency, which suggests that the enhancements and additions in the method are beneficial without compromising the practicality of the method.

On Dataset3, the overall metrics of all methods are noticeably lower than those on Dataset2, suggesting that this dataset is more challenging and more sensitive to small objects, dense distributions, or weak-texture scenarios. Under this setting, the proposed method still achieves the highest Precision and Recall, and is essentially on par with the best results in terms of mAP@50 and mAP@50-95, demonstrating robust generalization under more complex data distributions and stronger noise interference. This observation implies that the more direct benefits of MEMBA-F and CSCAF lie in improving detectability and stable recall on hard samples, i.e., enhancing the separability and visibility of weak targets through more sufficient cross-scale information interaction, thereby prioritizing the reduction of missed detections while maintaining high-confidence outputs. Meanwhile, the convergence of advantages on the stricter mAP@50–95 metric indicates that the upper bound of localization accuracy on Dataset3 is mainly constrained by annotation uncertainty, blurred target boundaries, or extreme scale variations. Even so, the proposed method maintains the best or jointly best overall accuracy without sacrificing inference efficiency, validating the robustness of the proposed architecture in balancing detection accuracy and deployment efficiency.

### 5.2 Ablation experimental results

To understand how each of these important modules helps in improving object detection of plant diseases and pests in real-world scenarios, ablation experiments are conducted, and their results are discussed in this section. As part of ablation experiments, we follow a standard detection framework with the same data, training, and evaluation protocols. In these experiments, we ablate each of these important modules, one at a time, by removing each of them from the entire model and also by combining both of them. As part of ablation experiments, we evaluate each of these modules by using metrics such as Precision, Recall, mAP@50, mAP@50-95, and latency. A summary of ablation experiments’ results is shown in **Table 6**.

**Table 6:** Ablation experimental results on dataset 1–3.

Dataset	Method	Params (M)	Precision	Recall	mAP@50	mAP@50-95	Inference speed (ms)
Dataset 1	YOLOv10	15.4	0.548	0.354	0.563	0.348	25.3
	+MEMBA-F	16.8	0.556	0.359	0.569	0.353	26.7
	+CSCAF	16.9	0.564	0.366	0.575	0.359	26.8
	Ours	17.2	**0.573**	**0.372**	**0.582**	**0.365**	27.4
Dataset 2	YOLOv10	15.4	0.648	0.602	0.576	0.392	25.3
	+MEMBA-F	16.8	0.664	0.621	0.592	0.404	26.7
	+CSCAF	16.9	0.678	0.637	0.603	0.413	26.8
	Ours	17.2	**0.691**	**0.652**	**0.614**	**0.421**	27.4
Dataset 3	YOLOv10	15.4	0.384	0.441	0.453	0.197	25.3
	+MEMBA-F	16.8	0.401	0.462	0.468	0.205	26.7
	+CSCAF	16.9	0.415	0.478	0.468	0.211	26.8
	Ours	17.2	**0.427**	**0.495**	**0.492**	**0.217**	27.4

Bold values indicates the optimal result.

As shown in **Table 6**, it can be seen that the performance of the method with the addition of the proposed MEMBA-F and CSCAF on all three datasets increases steadily compared with the baseline method YOLOv10, and the optimal performance is achieved when the proposed methods are used simultaneously. This demonstrates the effectiveness of the proposed methods when used independently. It is also seen that the addition of the proposed MEMBA-F method alone achieves optimal performance in terms of Precision, Recall, and mAP, demonstrating the effectiveness of the enhancement of the multi-scale features in the representation of weak texture lesions and small targets, resulting in fewer missed detections. It is also seen that the addition of the proposed CSCAF method alone achieves optimal performance in terms of Recall and mAP@50-95, demonstrating the effectiveness of the proposed cross-scale context-aware fusion method in reducing the influence of complex background information by aligning high-level semantic information with fine-granularity features. It is also seen that the optimal performance is achieved when the proposed method combines the advantages of the proposed methods, as all three datasets achieve the optimal performance in terms of Precision, Recall, mAP@50, and mAP@50-95. Furthermore, the experimental results were compared with those of other common modules, as shown in **Table 7**.

**Table 7:** Comparison with alternative feature enhancement modules on dataset 1–3.

Dataset	Method	Precision	Recall	mAP@50	mAP@50-95
Dataset 1	YOLOv10	0.548	0.354	0.563	0.348
	+Lightweight Conv	0.551	0.352	0.565	0.347
	+Depthwise Separable Conv	0.553	0.357	0.567	0.351
	+Local Attention	0.546	0.358	0.562	0.350
	**+MEMBA-F**	**0.556**	**0.359**	**0.569**	**0.353**
Dataset 2	YOLOv10	0.648	0.602	0.576	0.392
	+Lightweight Conv	0.651	0.598	0.580	0.390
	+Depthwise Separable Conv	0.646	0.607	0.573	0.395
	+Local Attention	0.658	0.616	0.586	0.400
	**+MEMBA-F**	**0.664**	**0.621**	**0.592**	**0.404**
Dataset 3	YOLOv10	0.384	0.441	0.453	0.197
	+Lightweight Conv	0.389	0.437	0.456	0.196
	+Depthwise Separable Conv	0.379	0.438	0.449	0.195
	+Local Attention	0.397	0.456	0.464	0.202
	**+MEMBA-F**	**0.401**	**0.462**	**0.468**	**0.205**

Bold values indicates the optimal result.

As shown in **Table 7**, replacing the neck enhancement component with simpler alternatives does not consistently improve performance across all datasets. Lightweight Conv and Depthwise Separable Conv slightly improve some indicators on Dataset1, but their gains are limited and even lead to performance degradation in several metrics on Dataset2 and Dataset3, indicating that purely local convolutional enhancement is insufficient for complex field scenes with weak textures and dense targets. Local Attention achieves relatively better results on Dataset2 and Dataset3, but it still remains inferior to MEMBA-F, suggesting that local contextual interaction alone cannot fully capture the spatially dispersed and cross-region dependencies of plant disease and pest instances. In contrast, MEMBA-F obtains the best Precision, Recall, mAP@50, and mAP@50–95 on all three datasets, demonstrating that the combination of local texture enhancement and selective long-range dependency modeling is more effective for small-target detection, weak lesion representation, and dense-instance recognition in complex agricultural environments.

### 5.3 Visualize experimental results

#### 5.3.1 Experimental results of loss function varying with epoch

**Figure 6:**
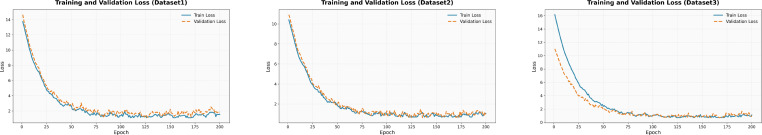
Experimental results of loss function varying with epoch.

As a way to visually understand the process of optimization and the convergence of the training, this section provides a casual exploration of the loss curve over the epochs. By tracking the average loss at each epoch and observing its movement over time, it is possible to see the learning process of the model, the rate of its convergence, and the stability of the backpropagation and the updates. This visualization also allows for the identification of problems such as oscillation, overfitting, and underfitting, as well as the determination of the number of epochs for training 16 the future. The results are presented in **Figure 6**.

As depicted in **Figure 6**, the losses, both training and validation, for all three datasets exhibit a very familiar optimization curve, which generally starts with a sharp decrease and gradually levels off to a steady convergence as the epochs increase. In the initial stages, the sharp decrease in the losses reflects the model’s quick learning of discriminative features. After that, the curve plateaus and remains steady at a low value, which reflects the refinement phase with stability. Throughout the process, the difference between the training and validation losses remains low, and they decrease almost together for most epochs, with no signs of divergence and ever-increasing validation losses, which reflects the model’s good generalization and low chances of overfitting. The minor fluctuations at the end are likely due to the mini-batch effects and data complexity, but they do not change the convergence pattern.

#### 5.3.2 Qualitative experimental results

**Figure 7:**
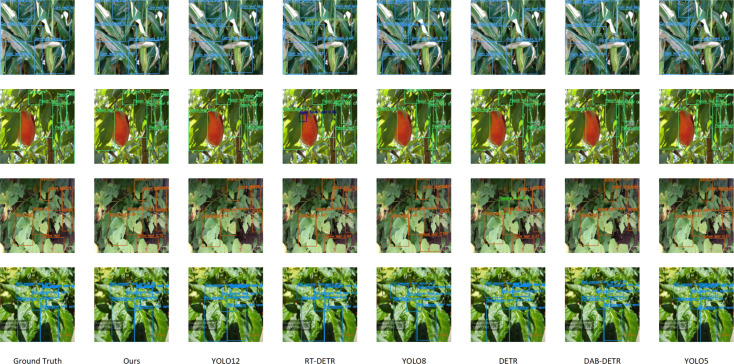
Qualitative detection results of dataset 1.

To better understand the actual performance of the model in the detection of plant diseases and pests, and to better comprehend the interpretability of the results, this section presents a qualitative assessment of the results obtained from the visualizations. By comparing the results obtained by the model with the ground truth, it is possible to better understand the performance of the model with regard to the detection of small objects, the management of crowding, the detection of weak texture lesions, and the management of the background. To better comprehend the performance of the model, the results obtained from the Dataset 1 are discussed, and the visualizations are presented in **Figure 7**.

From the visual comparison in **Figure 7**, it is obvious that various detection methods have different responses to challenging scenarios characterized by complex leaf texture, occlusion and small target size. Some methods in the baseline have issues with missing detections or incorrectly identifying background texture as target. Moreover, there are issues with consistent box position and size in crowded scenarios, characterized by shifted boxes, wrong box sizes, and even duplicates. However, in our method, there is a much closer alignment with ground truth boxes in various cases, consistent coverage of targets even in occluded and low-contrast scenarios, and better suppression of background noise. This is an indication of more consistent localization and fewer false alarms, implying that our method’s multi-scale feature enhancement and cross-scale fusion have a positive impact on detectability.

**Figure 8:**
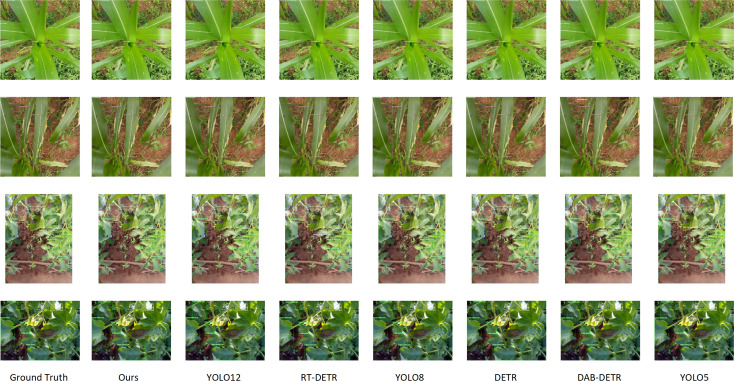
Qualitative detection results of dataset 2.

**Figure 9:**
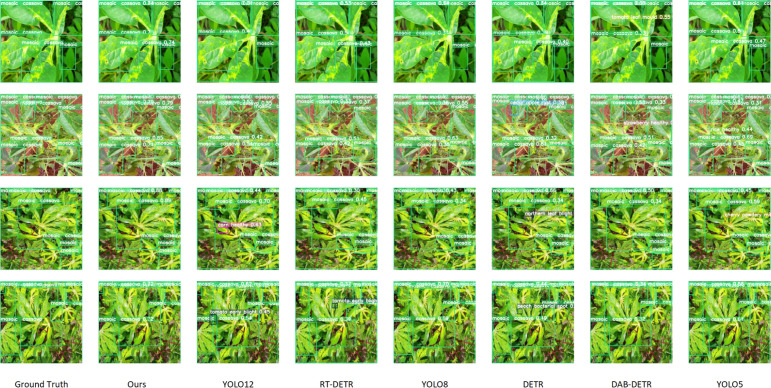
Qualitative detection results of dataset 3.

Furthermore, the qualitative experimental results for datasets 2 and 3 are presented, as shown in **Figures 8**, **9**.

From the visual comparisons of the above figures, it is clear that, with the natural scenes of Dataset2 and the complex, dense, and weakly textured scenes of Dataset3, the difference between methods in the detection of small lesions, occluded targets, and background interference is evident. Some of the methods have the drawback of missing detections, duplicate boxes, and the identification of textures such as leaf veins and shadows as disease targets. They also have the drawback of inconsistent box sizes and locations in the case of dense scenes, which causes the box to shift and the boundaries to be incomplete. However, the proposed method has the advantage of better coverage of the actual targets and fewer false positives in the background for all the examples. It has the added advantage of better stability and coherence in the localization and box size for the identification of tiny lesions and dense scenes, which makes the model robust and generalizable. This is in line with the workings of the proposed method. The multi-scale feature enhancement of the proposed MEMBA-F module makes the separation of weakly textured and low-contrast targets clearer, which reduces the chances of missing detection. The cross-scale context-aware fusion of the CSCAF module makes the identification of the relevant scene and the suppression of the irrelevant background clearer, which reduces the false positives and makes the localization stability better.

#### 5.3.3 Confusion matrix experimental results

**Figure 10:**
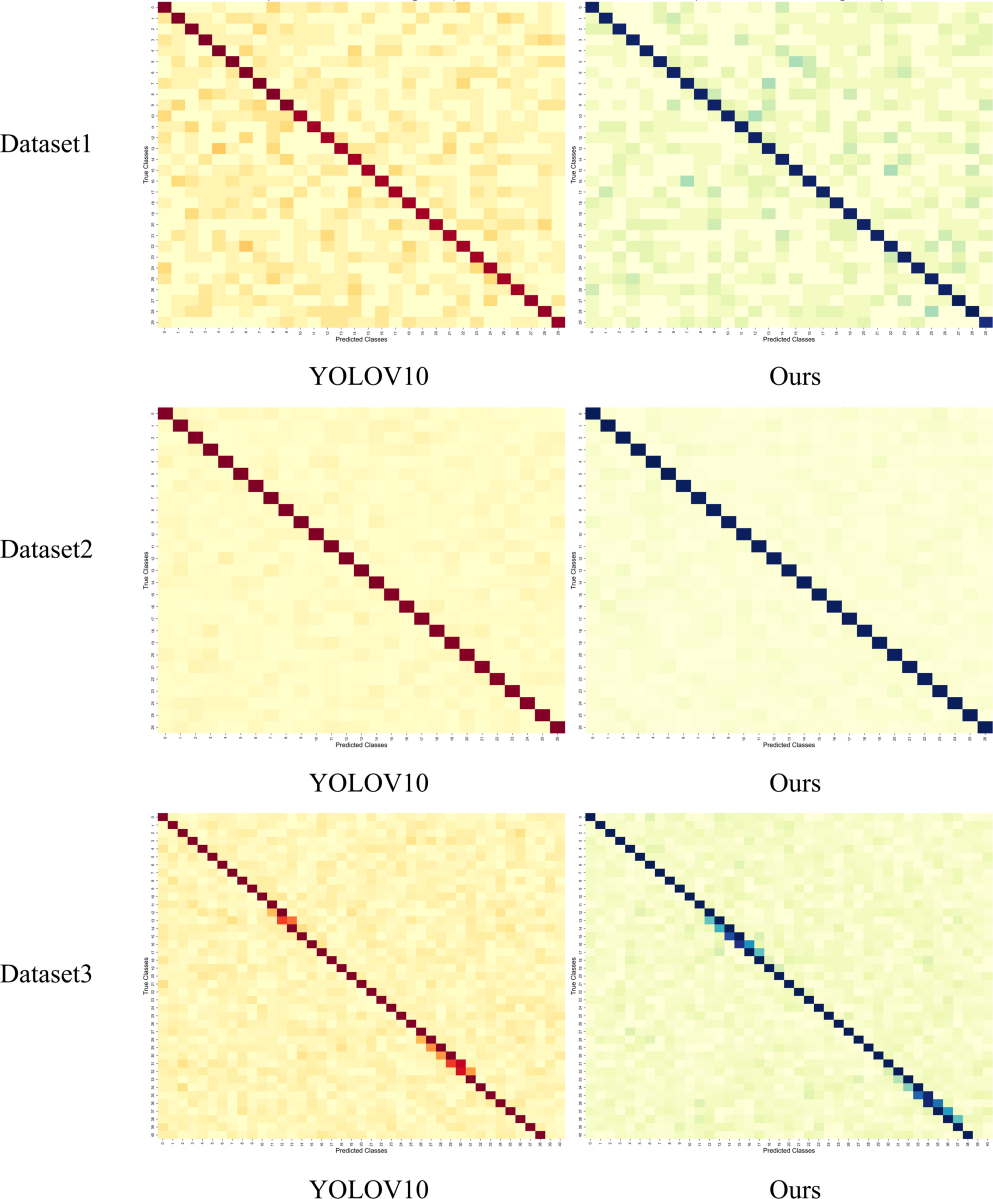
Confusion matrix experimental results.

As may be seen in **Figure 10** below, where the confusion matrices for Dataset1, Dataset2 and Dataset3 are provided, the YOLOv10 and the proposed method have a well-defined major diagonal. However, the proposed method also offers a tighter and more concentrated diagonal together with lighter off-diagonal activities. This is indicative of a higher rate of correct category prediction for most classes and a significant drop in cross-class misclassification and mutual confusion. This is to say that the model does not only improve the overall detection accuracy but also offers a more stable discriminative boundary between classes, making misclassification between visually similar lesion patterns and pest classes that look alike impossible.

In **Figure 10**, when observing Dataset1 and Dataset2, it is clear that both YOLOv10 and our method have a clear main diagonal line. However, it is also clear that our method has a tighter and cleaner diagonal line with lighter areas around it, which means that more accurate category information is provided for most of the classes, with fewer cross-class errors and mutual confusions between different classes. All these results suggest that, in addition to providing better accuracy for object detection, our method also provides a clear decision boundary between different classes, which means that visually similar lesion patterns or pest categories that look similar will not be confused with each other.

In terms of the confusion matrices in Dataset3, it is more challenging. However, the proposed method shows clearer off-diagonal blocks in the confusion matrices of YOLOv10. This indicates that the proposed method is more robust and generalized in complex background, weak texture, and crowded targets. This is consistent with the innovations of the proposed method. In terms of the innovations of MEMBA-F, it utilizes the multi-scale feature boost method. This method enhances the texture features of lesions, improves the consistency of each class, and limits the confusion caused by insufficient information. CSCAF utilizes the cross-scale context-aware fusion method. This method combines high-level semantic information and low-level detailed information. It also limits the confusion caused by background information. As a result, the proposed method is more stable, centralized, and dominant in the confusion matrices in all three datasets.

#### 5.3.4 Grad-Cam experimental results

**Figure 11:**
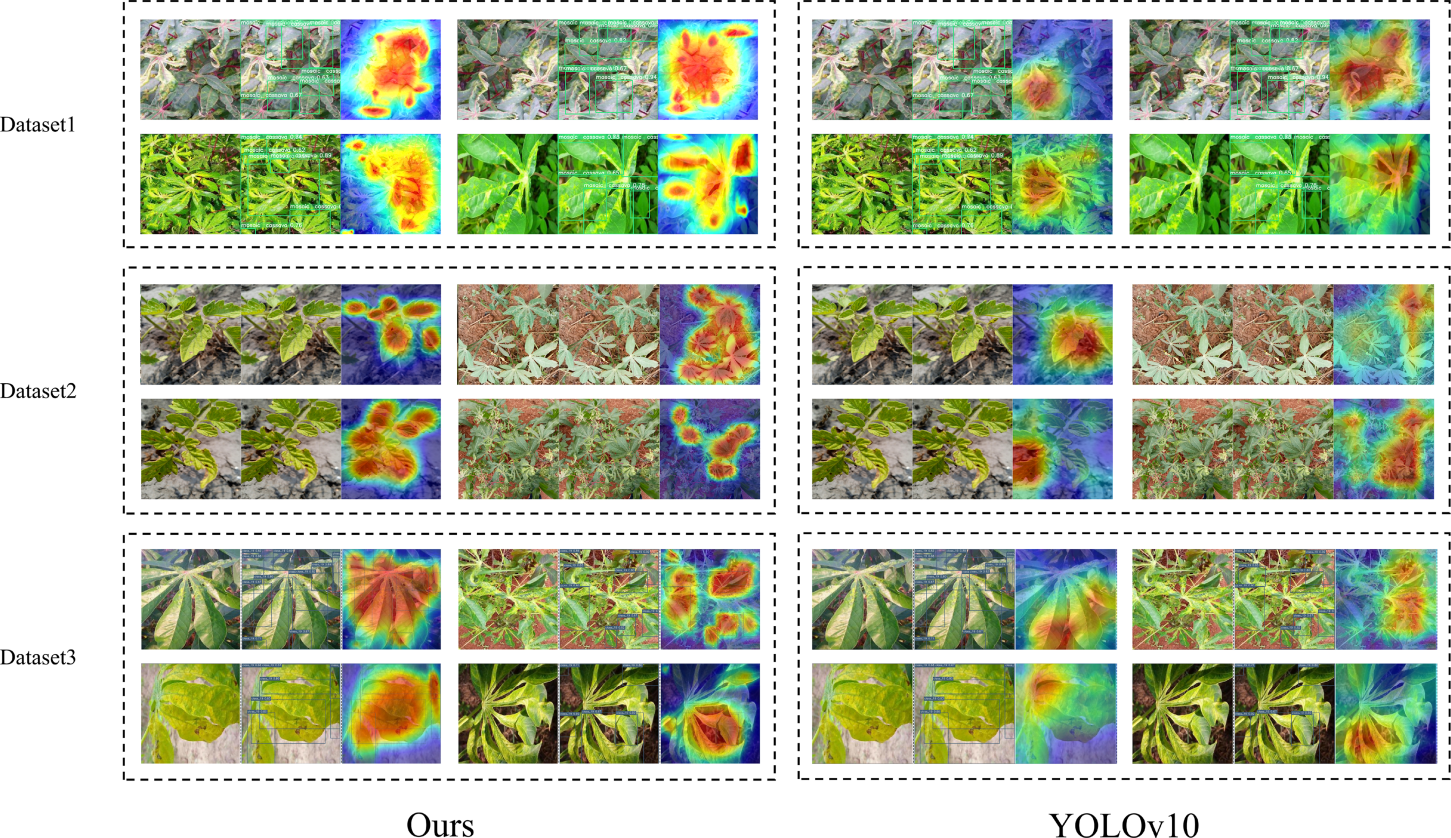
Experimental results of Grad-Cam comparing YOLOv10 and the proposed algorithm.

In order to further improve the way we comprehend the model’s decision-making and to visually check the focus of the model, the Grad-CAM visualization method is utilized for the exploration of the results. The Grad-CAM method generates a class activation heatmap based on the gradient information of the target class, focusing on the major areas of interest for the model during the decision-making process. This also provides insight into the way the model handles the boundaries of the lesions and the background noise, as well as the way the feature enhancement and fusion module works. The results are presented in **Figure 11**.

**Figure 11** presents the Grad-CAM visualization results comparing YOLOv10 and the proposed method on representative samples from Dataset1, Dataset2, and Dataset3. Compared with YOLOv10, the proposed method produces more concentrated and target-consistent activation regions, with high responses more clearly aligned with lesion or pest areas and their surrounding discriminative boundaries. In contrast, YOLOv10 shows more dispersed attention in several samples and is more easily affected by background textures such as leaf veins, shadows, soil, and non-diseased leaf regions. These visualization results indicate that the proposed MEMBA-F and CSCAF modules help the model enhance weak disease or pest responses and suppress irrelevant background interference. Specifically, MEMBA-F improves the salience of small and weak-texture targets through multi-scale feature enhancement, while CSCAF strengthens the consistency between semantic cues and fine-grained localization details through cross-scale context fusion. Therefore, the Grad-CAM results further support the effectiveness and interpretability of the proposed method in complex plant disease and pest detection scenarios.

### 5.4 Counting performance under different density buckets

**Figure 12:**
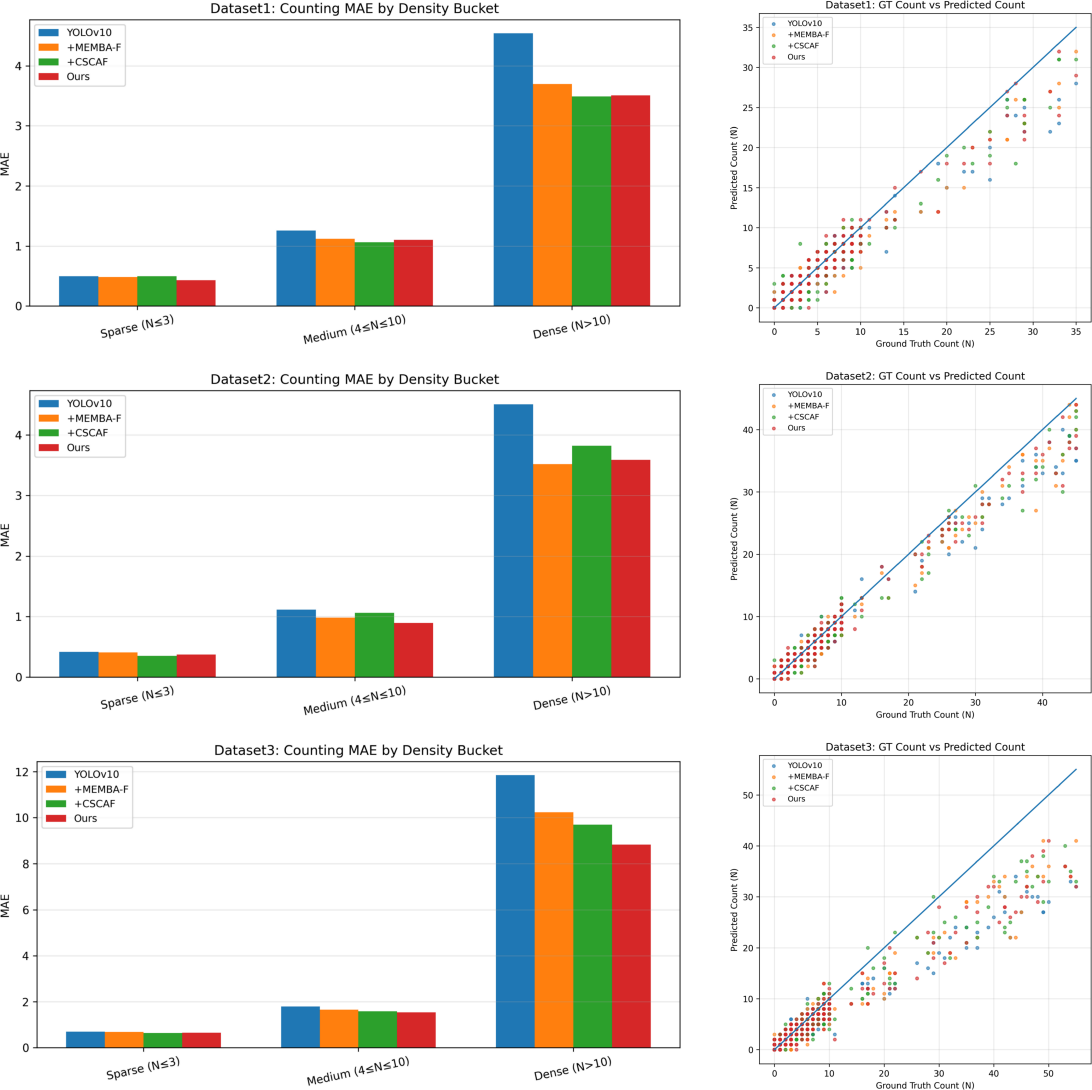
Counting performance comparison under sparse/medium/dense density buckets on dataset 1–3, reporting MAE bars and GT-versus-predicted count scatter plots for different methods.

To validate the effectiveness and robustness of the proposed method for plant disease and pest object counting, this section further conducts a density-binned counting evaluation, where the number of detected instances is used as the counting result. Specifically, let the ground-truth number of objects in the *i*-th image be *N_i_*, and the predicted number be ${\hat{N}}_{i}$. According to *N_i_*, the test samples are divided into three density intervals: Sparse (*N_i_*≤ 3), Medium (4 ≤ *N_i_*≤ 10), and Dense (*N_i_ >* 10). Within each density interval, the mean absolute error (MAE) is computed to quantify the counting bias and error variability under different object densities. In addition, a scatter plot of $\hat{N}$ versus *N* together with the reference line *y* = *x* is drawn to intuitively characterize the consistency between the predicted counts and the ground-truth numbers. The figure presents the MAE comparison of different methods across the three density intervals on the three datasets, as well as the corresponding *N* versus $\hat{N}$ scatter consistency distributions. This enables a comprehensive assessment of counting stability and deployability in sparse, small-scale, and dense scenarios. The results are shown in **Figure 12**.

As shown in the figure, across different density bins on the three datasets, the counting error increases with object density. The Dense bin (*N >* 10) is the most challenging setting for all methods. However, the proposed method consistently achieves lower MAE in the Dense bin than YOLOv10 and the two single-module variants, and its scatter distribution lies closer to the reference line *y* = *x*, indicating more stable counting consistency and smaller systematic bias even under dense small-target and complex-background conditions. This is consistent with what the modules are designed to do. The feature enhancement of weak texture lesions and small insects through multi-scale feature enhancement of MEMBA-F reduces the problem of undercounting due to undetected objects. On the other hand, the cross-scale context fusion of CSCAF combines high-level meaning and detailed objects, reducing the problem of overcounting due to double detections and false positives. These modules enable the model to provide more dependable counts, ranging from sparse to dense scenes. In agricultural scenes, dependable object counting provides robust data for assessing the density of pests, grading the severity of diseases, and making decisions regarding the administration of pesticides and other management practices. In high-density outbreak situations, for example, this combo reduces management risks arising from undetected reports and false positives, making precision plant protection more efficient.

## 6 Conclusion

This paper focuses on the detection and counting of plant diseases and pests. Our method is based on the YOLOv10 architecture and includes the addition of the Neck module, consisting of the MEMBA-F module for multi-scale feature enhancement and the CSCAF module for cross-scale context-aware fusion. The method achieves better Precision, Recall, and mAP@50, as well as mAP@50–95 on all three datasets, with consistent performance gains without compromising the speed of the model. The visualizations also demonstrate that the proposed method can focus more accurately on disease and pest target regions while reducing interference from background noise. The density-based counting also shows the accuracy of the method, with lower errors and higher consistency, thus indicating its potential value for field-oriented disease and pest monitoring rather than confirming completed deployment in real agricultural machinery or edge-device systems.

In future, it is expected that the scope of future works will be widened in terms of data, model, and application dimensions. In terms of data, it is expected that larger and more diverse data will be included, which will cover different regions, crops, and imaging conditions. Moreover, active learning and weakly supervised annotation will also be applied to reduce costs associated with data collection and annotation while improving generalization for rare categories and challenging scenarios like extreme lighting or occlusion. In terms of the model, it is expected that tighter joint learning will be applied for better performance in terms of interpreting results for dense scene counting. In terms of application, future work will further evaluate the deployment feasibility of the model on mobile or edge devices, including hardware-specific inference speed, memory usage, computational power, and energy consumption. On this basis, the model may be further combined with continuous temporal monitoring for trend analysis and early warning of disease and pest outbreaks, and may also provide technical support for future integration with farmland management systems toward a more complete precision plant protection workflow.

## Data Availability

The original contributions presented in the study are included in the article/supplementary material. Further inquiries can be directed to the corresponding authors.
