# Correction: A study on plant disease and pest detection and counting based on multi-scale enhancement and cross-scale fusion

**DOI:** 10.3389/fpls.2026.1927536

**Published:** 2026-07-13

**Authors:** Frontiers Production Office

**Affiliations:** Frontiers Media SA, Lausanne, Switzerland

**Keywords:** cross-scale attention fusion, multi-scale feature enhancement, object counting, plant disease and pest detection, precision agriculture

An equal contributions statement was not removed from authors “Junshu Wang and Li Liang” during production, in error. The city name for affiliation 3 “Shenzhen International Graduate School, Tsinghua University” has been corrected from “Nanning” to “Shenzhen”. The institution “School of Computer Science” has been removed from Affiliations “4 and 5”.

The original version of this article has been updated.

